# Biopreservation of Wild Edible Mushrooms (*Boletus edulis*, *Cantharellus*, and *Rozites caperata*) with Lactic Acid Bacteria Possessing Antimicrobial Properties

**DOI:** 10.3390/foods11121800

**Published:** 2022-06-18

**Authors:** Elena Bartkiene, Egle Zokaityte, Vytaute Starkute, Ernestas Mockus, Dovile Klupsaite, Justina Lukseviciute, Alina Bogomolova, Audrone Streimikyte, Fatih Ozogul

**Affiliations:** 1 Department of Food Safety and Quality, Veterinary Academy, Lithuanian University of Health Sciences, Tilzes Str. 18, LT-47181 Kaunas, Lithuania; egle.zokaityte@lsmuni.lt (E.Z.); vytaute.starkute@lsmuni.lt (V.S.); justina.lukseviciute@stud.lsmu.lt (J.L.); alina.bogomolova@stud.lsmu.lt (A.B.); audrone.streimikyte@stud.lsmu.lt (A.S.); 2 Institute of Animal Rearing Technologies, Faculty of Animal Sciences, Lithuanian University of Health Sciences, Tilzes Str. 18, LT-47181 Kaunas, Lithuania; ernestas.mockus@lsmuni.lt (E.M.); dovile.klupsaite@lsmuni.lt (D.K.); 3 Department of Seafood Processing Technology, Faculty of Fisheries, Cukurova University, 01330 Adana, Turkey; fozogul@cu.edu.tr

**Keywords:** edible mushrooms, fermentation, lactic acid bacteria, volatile compounds, biogenic amines

## Abstract

There is scarce data on the influence of fermentation with lactic acid bacteria (LAB) on the quality and safety of edible mushrooms. The aim of this study was to ferment *Suillus luteus*, *Boletus edulis*, *Cantharellus cibarius*, and *Rozites caperata* with LAB strains (*Lacticaseibacillus casei* LUHS210 and *Liquorilactobacillus uvarum* LUHS245) and to evaluate the influence of this technology on colour characteristics, pH, mould/yeast count, liking, emotional response, volatile compound (VC) profile, and the formation of biogenic amines (BA). Additionally, ultrasonication or prolonged thermal treatment were applied before fermentation. The LUHS245 strain showed better preservation properties in the case of fungal inhibition; however, prolonged thermal treatment and/or ultrasound pre-treatment ensure safer fermentation. Mushroom species and type of pre-treatment had a significant effect on colour coordinates and pH (*p* ≤ 0.0001). A greater variety of VC was identified in pre-treated and fermented samples. Significant differences were found between the emotions induced in consumers. The lowest sum of BA was found in thermally pre-treated and fermented *R. caperata,* while the highest was in ultrasonicated and fermented *B. edulis*. Finally, despite good overall acceptability, it is important to select appropriate LAB strains for the fermentation of edible mushrooms to ensure their safety in the case of BA formation.

## 1. Introduction

Wild edible mushrooms have been a part of the human nutrition culture for many years because of their sensory characteristics, good nutritional and functional characteristics, and attractive culinary characteristics [1]. Mushrooms are a good source of complex and non-digestible carbohydrates; for this reason, they can act as prebiotics [2]. Taking into consideration their valuable compounds, such as high-biological-value proteins, vitamin D, β-glucans, minerals, unsaturated fatty acids, phenolic compounds, ergosterols, tocopherols, lectins, and dietary fibres, mushrooms become an attractive functional food [3]. Moreover, mushrooms contain a low level of fat, carbohydrates, sodium, and calories and they are cholesterol-free [1]. As edible mushrooms are the most diverse and ecologically and economically important species in the world, in order to maintain their post-harvest quality, new preservation techniques, or a combination of them, have been sought [4]. Although thermal (cooling and drying), chemical (washing with antimicrobial and anti-browning agents, coating, and ozone), and physical (irradiation, plasma, packaging, and pulsed electrical field) preservation techniques, as well as long-term preservation (marinating with acetic acid, heavy salting, and freezing), significantly increase mushroom shelf-life, these methods have some limitations in maintaining mushroom quality and safety. [5,6]. Furthermore, some of these preservation techniques may have drawbacks such as a high initial investment or a lengthy processing time. Until now, in many countries with different nutrition traditions, lactic acid fermentation has been traditionally applied to preserve both wild-grown and cultivated edible mushrooms [6]. Compared to the salting process of mushrooms, fermentation with lactic acid bacteria (LAB) requires a lower salt content and significantly lower losses of nutrients could be obtained [6]. Lactic acid fermentation is a natural, simple, and widely available method for food processing. This method is also advantageous from an economic point of view; however, it has not been applied on a large industrial scale for mushroom preservation [6,7]. Microorganisms involved in the fermentation process belong to the group of LAB, which have a GRAS (Generally Recognised as Safe) status [8]. Moreover, the use of selected LAB strains with preservative characteristics provides beneficial properties for fermented foods: enrichment with vitamins, increased resistance to microbial spoilage, improved sensory characteristics, nutritional value, health-promoting effects, etc. [8,9,10,11]. The *Lacticaseibacillus casei* LUHS210 and *Liquorilactobacillus uvarum* LUHS245 used in this study were isolated from spontaneous rye sourdough and, as our previous studies showed, these LAB strains can be effective antimicrobial agents in food preservation [12]. However, despite the formation of desirable compounds during fermentation with LAB, non-desirable changes can also occur, i.e., the formation of biogenic amines (BA), the presence of which in foods is related to both food quality and safety [13]. In fermented foods, LAB, as well as other decarboxylase positive bacteria and yeasts, are able to produce BA through the decarboxylation of free amino acids [14,15]. A small amount of BA is always found in fermented food; however, if a large amount of these compounds accumulates in food and is consumed by humans, this could lead to serious health problems such as diarrhoea, vomiting, heart palpitations, and headaches [16]. Precursor availability, the presence of decarboxylase-positive microorganisms, and various intrinsic, technological, and environmental factors could influence BA accumulation in food [14]. Therefore, it is essential to select the appropriate starter LAB strain for mushroom fermentation in order to minimise BA production. Usually, edible mushrooms are thermally treated for a long time before fermentation; this process can reduce the concentration of their bioactive compounds, as well as decrease their functional and nutritional value. For this reason, ultrasound treatment, as a low-temperature technology, could be a better solution for the pre-treatment of mushrooms before fermentation to avoid the loss of bioactive compounds.

Commercially used preservation techniques for mushrooms still have some limitations in terms of product quality and economic efficiency. Therefore, further improvement in the quality of preserved mushrooms by applying a sustainable biological approach could gain more attention. The popularity of fermented foods is increasing due to their beneficial health-related attributes and attractive sensory properties. As studies on mushroom fermentation with LAB are scarce, there is a need to analyse the influence of this preservation method on the quality and safety of mushrooms. Therefore, in this study, we hypothesised that the use of selected LAB strains for the biopreservation of wild edible mushrooms could lead to better sensory properties of the final product because of the formation of different volatile compounds, and the safest LAB strain could be selected in accordance with the lowest concentration of BA. For this study, some of the most popular edible wild mushrooms in Central-Eastern Europe were selected: *Suillus luteus*, *Boletus edulis*, *Cantharellus cibarius*, and *Rozites caperata*. Preserving these mushrooms for an extended period of time is needed because they grow only in summer and autumn for a short time and have specific beneficial characteristics for human nutrition. *S. luteus* possess high antioxidant activity, but their extracts have low antimicrobial activity [17]. *B. edulis* mushrooms are a good source of bioactive molecules, including polyunsaturated fatty acids and phenolic compounds [18]. *C. cibarius* are rich in potassium and vitamins D and C [19] and show desirable properties such as antioxidant, antimicrobial, antihypertensive, immunomodulatory, antiviral effects, etc. [20]. *R. caperata* compounds are characterised by their antiviral activity [21]. Therefore, the aim of this study was to apply *Lacticaseibacillus casei* LUHS210 and *Liquorilactobacillus uvarum* LUHS245 strains for the fermentation of edible mushrooms (*S. luteus*, *B. edulis*, *C. cibarius*, and *R. caperata*) and to evaluate the influence of the technology used on the colour characteristics, pH, mould/yeast count, liking, emotional response, volatile compound profile, and formation of biogenic amines. Additionally, ultrasonication or prolonged thermal treatment were applied before fermentation.

## 2. Materials and Methods

### 2.1. Edible Mushrooms and LAB Strains Used for Fermentation

**Figure 1:**
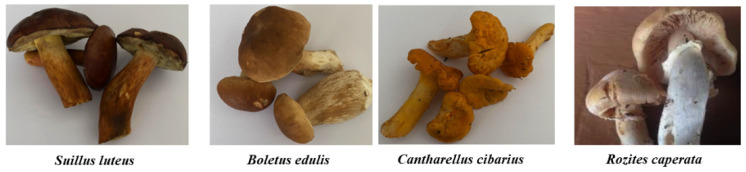
Images of edible mushrooms used in this experiment.

*S. luteus*, *B. edulis*, *C. cibarius*, and *R. caperata* mushrooms were collected in the Varenos Forests (Eastern Lithuania) in September of 2021. Images of the mushrooms are given in Figure 1. Fresh fruiting bodies were cleaned and boiled in water (1/1; mushroom mass by water volume) for 5 min with 3% salt (the concentration of salt was calculated from the fresh mushroom mass) [6]. After boiling, mushrooms were cooled to room temperature (22 ± 2 °C) and used for fermentation. Unfermented and unboiled mushrooms were analysed as control 1; boiled unfermented mushrooms were analysed as control 2. In total, 12 samples of mushrooms were prepared.

**Figure 2:**
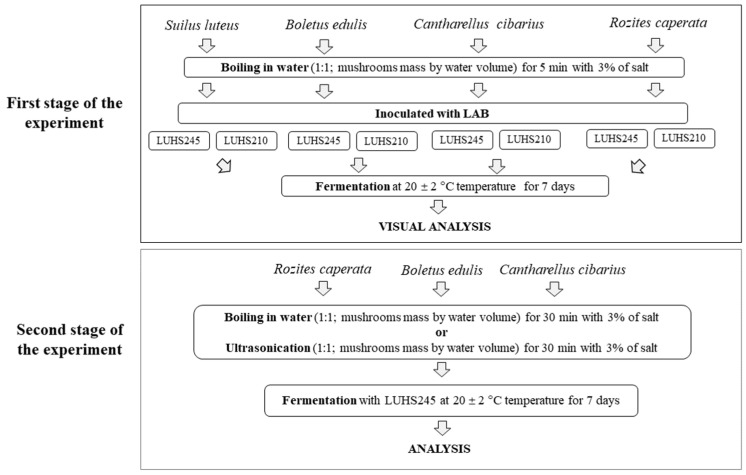
Principal scheme of the experiment (LUHS210—fermented with *L. casei* LUHS210; LUHS245—fermented with *L. uvarum* LUHS245).

Selected LAB strains were previously isolated from spontaneously fermented cereal, identified and characterised by Bartkiene et al. [12]. These LAB showed a broad spectrum of antimicrobial properties [12]. *L. casei* LUHS210 and *L. uvarum* LUHS245 strains were incubated separately and multiplied in De Man, Rogosa, and Sharpe (MRS) broth culture medium (Biolife, Milan, Italy) at 30 °C under anaerobic conditions. Boiled mushrooms (100 g) were inoculated with 3 mL of LAB multiplied in MRS (average cell concentration 9.0 log_10_ CFU/mL) followed by fermentation for 7 days at 22 ± 2 °C. The principal scheme of the first stage of the experiment is shown in Figure 2.

Further, mushroom samples were visually evaluated to determine which treatment led to the lowest intensity of visible mould colonies on samples’ surfaces. Then, the most effective LAB strain was selected for further treatment. Because of the dark unacceptable colour of *S. luteus* mushrooms, they were not used for subsequent experiments.

The parameters of thermal treatment and ultrasonication before fermentation were optimised. Ultrasonication was performed at a 37 kHz frequency, using a 100 W power level. The equipment employed was an ultrasonication processor (PROCLEAN 3.0DSP, Ulsonix, Berlin, Germany). Each sample was processed for 30 min. The principal scheme of the second stage of the experiment is shown in Figure 2.

### 2.2. Analysis of the Mushrooms’ Colour Characteristics, pH, and Mould/Yeast Count

Before measuring the mushrooms’ colour characteristics and pH, samples were homogenised using a blender.

The colour coordinates (L*, a*, b*) were assessed using a CIELAB system (Chromameter CR-400, Konica Minolta, Tokyo, Japan).

The pH was measured using a pH electrode (PP-15, Sartorius, Goettingen, Germany). The dry matter of the samples was evaluated with a saccharometer (Merck KGaA, Darmstadt, Germany).

Yeasts and fungi in edible mushrooms were counted on chloramphenicol agar (CM0549, Oxoid, UK) after incubation at 25 °C for 5 days. All experiments were carried out in triplicate, and the number of microorganisms was expressed as log_10_ of colony-forming units per gram (log_10_ CFU/g).

### 2.3. Determination of the Mushrooms’ Volatile Compounds (VC)

The VC in edible mushrooms were analysed by gas chromatography–mass spectrometry (GC-MS); we used a method with our own developed modification. A solid phase micro-extraction (SPME) device with Stableflex™ fibre coated with a 50 µm PDMS-DVB-Carboxen™ layer (Supelco, Inc., Bellefonte, PA, USA) was used for analysis. Before the experiment, mushroom samples were homogenised using a blender. For headspace extraction, 1 g of homogenised mushrooms were added to the 20 mL extraction vial which was sealed with polytetrafluoroethylene septa and thermostatted at 60 °C for 15 min. The fibre was then exposed to the headspace of the vial for 10 min and desorbed in the injector liner for 2 min (splitless injection mode). Prepared samples were analysed with a GCMS-QP2010 gas chromatograph (Shimadzu Corporation, Kyoto, Japan) and a mass spectrometer. The following conditions were used for analysis: injector temperature 250 °C, ion source temperature 220 °C, and interface temperature 280 °C. Helium (99.999% detector purity; AGA, Vilnius, Lithuania) was used as a carrier gas at a flow rate of 0.97 mL/min. The Rxi^®^-5MS capillary column (0.25 mm ID, 0.25 μm film thickness, 30 m length; Restek, Bellefonte, PA, USA) was used for analysis. The temperature gradient was programmed from a start at 35 °C (5 min hold) to 200 °C (10 °C/min) up to 280 °C (25 °C/min, 5 min hold). The VC were identified according to mass spectra libraries (NIST11, NIST11S, and FFNSC2).

### 2.4. Evaluation of the Overall Acceptability and Emotions Induced in Consumers by the Edible Mushrooms

The overall acceptability of the mushroom samples was established by 20 judges, using a 10-point scale ranging from 0 (‘dislike extremely’) to 10 (‘like extremely’) [22]. Samples were also tested by applying FaceReader 8.0 software (Noldus Information Technology, Wageningen, The Netherlands) and scaling eight emotion patterns (neutral, happy, sad, angry, surprised, scared, disgusted, and contempt) according to the procedure described by Bartkiene et al. [22].

### 2.5. Analysis of Biogenic Amine (BA) Content in Fermented and Untreated Edible Mushrooms

Sample preparation and determination of BA in mushroom samples were performed according to the method of Ben-Gigirey et al. [23], with some modifications described by Bartkiene et al. [24]. Before the experiment, mushroom samples were homogenised using a blender.

### 2.6. Statistical Analysis

Results were expressed as the mean ± standard deviation (SD). Thermal treatment, ultrasonication, and fermentation of mushrooms were performed once; all analyses were performed in triplicate. Results were analysed using the statistical package SPSS for Windows V15.0 (SPSS Inc., Chicago, IL, USA, 2007). A linear Pearson’s correlation was used to quantify the strength of the relationship between pH, mould/yeast count, biogenic amines, volatile compounds, overall acceptability, and emotional response. The correlation coefficients were calculated using the statistical package SPSS for Windows V15.0 (SPSS Inc., Chicago, IL, USA, 2007). Results were recognised as statistically significant at *p* ≤ 0.05.

## 3. Results and Discussion

### 3.1. Selection of the Most Appropriate LAB Strain for Edible Mushroom Fermentation

**Figure 3:**
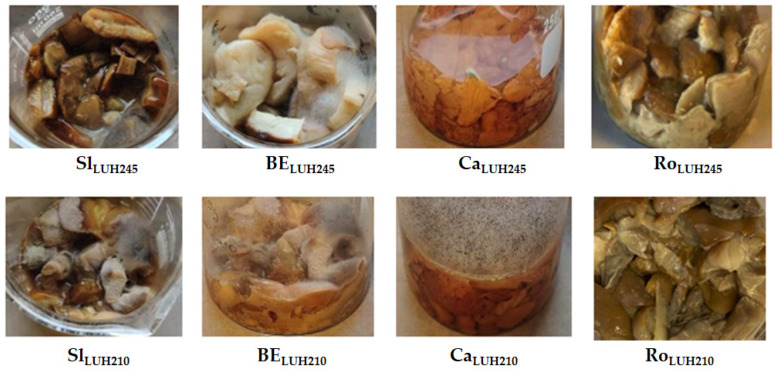
Images of edible mushrooms fermented for 7 days with *L. uvarum* LUHS245 and *L. casei* LUHS210 (Sl—*Suillus luteus*; BE—*Boletus edulis*; Ca—*Cantharellus* *cibarius*; Ro—*Rozites caperata*).

Images of the fermented mushrooms are given in Figure 3. According to the intensity of visible mould colonies on the surface of samples, *L. uvarum* LUHS245 was selected for the subsequent experiment. Prolonged thermal treatment and ultrasonication pre-treatment were suggested to avoid the moulding of mushrooms. *S. luteus* was not used for subsequent experiments because of its dark unacceptable colour.

**Figure 4:**
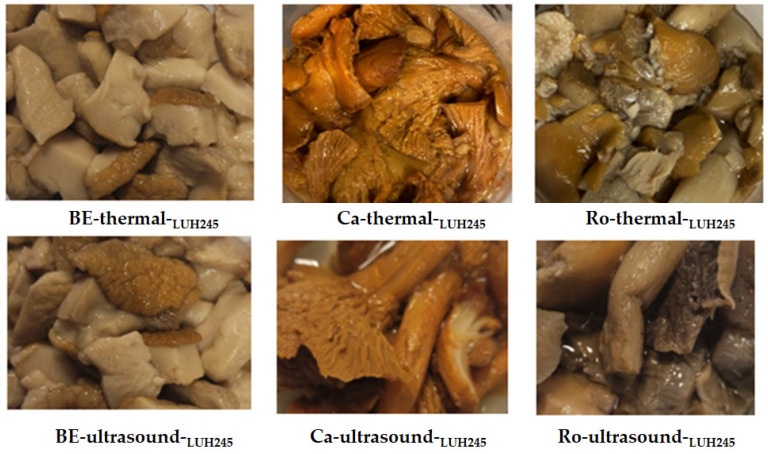
Images of edible mushrooms fermented for 7 days with *L. uvarum* LUHS245 (BE—*Boletus edulis*; Ca—*Cantharellus*; Ro—*Rozites caperata*).

Images of the pre-treated (boiled and ultrasonicated) mushrooms fermented with *L. uvarum* LUHS245 are given in Figure 4. After prolonged thermal treatment (30 min) and ultrasonication (30 min), no mould colonies were visible on the surface of the samples.

For the subsequent experiments, both thermal treatment and ultrasonication of mushrooms, and fermentation with *L. uvarum* LUHS245 were used.

Food spoilage caused by fungi represents a big concern not just for economic losses, but also for its risk to human health. Some of the compounds excreted by LAB possess antifungal properties, so various LAB are used for food preservation. In this study, *L. casei* LUHS210 and *L. uvarum* LUHS245, originally isolated from fermented cereals, showed good antifungal activity against *Aspergillus nidulans*, *Penicillium oxalicum*, *Penicillium funiculosum*, *Fusarium poae*, and *Fusarium graminearum* [12]. In most of the reported studies, the antifungal activity of LAB is explained by different amounts of organic acids excreted into the supernatants. However, according to Magnusson et al. [25], there are some cyclic dipeptides excreted by LAB that inhibit the sporulation of fungi. In this study, *L. uvarum* LUHS245 was used for the subsequent experiment according to the intensity of visible mould colonies on the surface of the samples.

### 3.2. Colour Characteristics, pH, and Mould and Yeast Count in Edible Mushrooms

The colour characteristics and pH of the edible mushrooms are given in Table 1. Comparing the colour parameters of thermally treated and ultrasonicated unfermented mushrooms of the same species, thermally treated *B. edulis* samples, showed higher redness (a*) and yellowness (b*) than those of ultrasonicated samples (13.9% and 8.8% higher, respectively). However, higher coordinates of lightness (L*) were found for the ultrasonicated *B. edulis* and *R. caperata* samples than for thermally treated samples (1.92% and 15.5% higher, respectively). Ultrasonicated *Cantharellus* and *R. caperata* samples showed higher a* and b* coordinates than those of thermally treated samples of the same species (a*– 53.9% and 40.6% higher, respectively; b*—30.6% and 29.3% higher, respectively). There were no significant differences in the pH of thermally treated and ultrasonicated unfermented mushrooms of the same species; the highest pH was found in *Cantharellus* mushrooms (on average, 18.5% higher than in *B. edulis* and *R. caperata*). Comparing the colour coordinates of thermally treated and ultrasonicated fermented mushrooms, ultrasonicated fermented *B. edulis* and *R. caperata* showed higher L*, and ultrasonicated fermented *Cantharellus* and *R. caperata* showed higher a* and b* coordinates than those of thermally treated and fermented samples of the same species (L*—3.13% and 7.20%, a*—11.3% and 4.26%, b*—4.39% and 3.71% higher, respectively). While comparing the pH of thermally treated and ultrasonicated fermented samples, a significantly lower pH was found in thermally treated and fermented *R. caperata* samples than in ultrasonicated and fermented ones. However, no significant differences were established between other mushroom samples. Multivariate analysis of variance showed that mushroom species, thermal treatment and/or ultrasonication, and the interaction of these factors had a significant effect on the samples’ colour coordinates and pH (*p* ≤ 0.0001). In comparing the mould and yeast count in thermally treated and ultrasonicated unfermented mushrooms of the same species, a significantly lower mould and yeast count was found in thermally treated unfermented *R. caperata* samples; however, no significant differences were established between thermally treated and ultrasonicated fermented mushroom samples of the same species. When comparing thermally treated and ultrasonicated mushrooms with fresh samples, a lower mould and yeast count were established in treated samples in all cases, and a significant moderate positive correlation (r = 0.6430, *p* = 0.0001) was found between the samples’ pH and mould and yeast count.

The colour of mushrooms is one of the most important indicators of their quality, affecting consumers’ purchase intentions. The darkening of the mushroom surface is mainly related to microbial contamination, enzymatic activity, or mechanical injuries [26]. Lactic acid fermentation influences the colour of mushrooms, and this is mainly related to pre-treatment procedures such as blanching [6]. According to Jabłońska-Ryś et al. [27], the stabilisation of the L* value and the reduction in the a* value could be observed during the LAB fermentation of mushrooms, while the b* value increases at every stage of this technological process (washing, blanching, and fermentation). Liu et al. [28] reported the decrease in the L* value and the increase in the values of a* and b* of oyster mushrooms fermented for 18 days. In the field of food technology, ultrasound is used for preservation, processing, and extraction. It induces physical and chemical changes in food matrices and could diminish damage or prolong the storage time of foods [29]. However, the influence of ultrasonication on food quality is related to the treatment conditions. The discolouration of white mushrooms could slow down after ultrasound treatment because of the reduction of peroxidase, polyphenol oxidase, and other enzyme activities involved in respiratory pathways [30]. This could explain the higher coordinates of lightness in ultrasonicated and ultrasonicated fermented *B. edulis* and *R. caperata* found in this study. Thermal treatment could also lead to the loss of desirable food compounds, including colour pigments [31]. Thermal pre-treatment of mushrooms enhances the value of the red colour (a*) as non-enzymatic browning [32]. In our study, this was also observed with thermally treated *B. edulis* samples.

The production of organic acids by LAB causes a reduction in pH and reflects the progress of fermentation. It is known that fermented product with a pH of 4.0–4.1 is durable [6]. In the present study, the pH of most fermented samples was within this range. Moreover, a lower number of yeasts and moulds were detected in pre-treated or/and fermented mushrooms, compared to fresh ones. This could be explained by the thermal pre-treatment and ultrasonication contributing to the partial reduction in microflora [33]. Moreover, organic acids generated during fermentation are a major safeguard against food spoilage microorganisms, since their undissociated forms exhibit strong microbial antagonistic effects. Comparing the antimicrobial potential of different organic acids, acetic acid inhibits yeasts and moulds more than lactic acid [12,34,35,36]. Since antifungal activity is mainly related to the production of acetic rather than lactic acid (because of acetic acid’s higher dissociation constant), heterofermentative LAB display a wider spectrum of antifungal activity in comparison with homofermentative ones [37,38]. 

### 3.3. VC Profile of Mushrooms

The whole VC profile of the untreated and treated mushrooms is given in Supplementary Materials (Table S1. Volatile compound profile). The VC in fresh mushrooms (% of total VC for those accounting for >1% of the whole profile) is shown in Table 2. The main VC in all the tested fresh mushrooms was 1-octen-3-ol, which was the highest in the *B. edulis* VC profile (61.9%). The other most abundant VCs were 3-octanol and 1-octanol in *B. edulis*, oct-(2E)-enal and (E)-2-octen-1-ol in *C. cibarius*, and benzaldehyde in *R. caperata*.

Mushroom flavour is mainly formed by the aliphatic components, such as 1-octen-3-ol, 2-octen1-ol, 3-octanol, 1-octanol, and 3-octanone [39]. Compounds, such as fatty acid degradation products, terpenoids, 3-(methylthio)propanal, and *N*-heterocyclic compounds cause individual odours between different mushroom species [40]. The unsaturated alcohol, 1-octen-3-ol, is very common in most mushroom species and is characterised as a “mushroom-like flavour” and “raw mushroom”. The odour of 3-octanol is described as earthy, mushroom, dairy, creamy dairy, musty, creamy, waxy with a slight fermented green minty nuance, herbaceous, somewhat nutty, and fatty, and the taste is musty, mushroom, and earthy; 1-octanol possesses a medium-strength waxy, green, citrus, aldehydic, and floral odour with a sweet, fatty, coconut nuance, and a waxy, green, citrus, orange, and aldehydic taste with a fruity nuance; the odour of oct-(2E)-enal is described as fresh, cucumber, fatty, green, herbal, banana, waxy, green, and leafy, and the taste is described as sweet, green, citrus, peel, spicy, cucumber, oily, fatty, and brothy; the odour of (E)-2-octen-1-ol is green and vegetable-like, lending a pleasant, fatty note to fruity complexes, and its taste is strong, fatty, oily, and sweet, with fruity and citrus notes [41]. Zhuang et al. [42] found that the main VC in four boletus varieties were aldehydes, esters, alcohols, acids, pyrazines, ketones, and phenols; 1-octen-3-ol and 2,5-dimethylpyrazine were the predominant VC among samples, while 3-(methylthio)propionaldehyde and 2,6-dimethylpyrazine were the main compounds in *B. edulis*.

Comparing the VC profile of fresh mushrooms with thermally treated and ultrasonicated ones, a greater variety of VC was identified in treated mushrooms (Table 3). Comparing the changes of VC in thermally treated and ultrasonicated mushroom samples of the same species, in thermally treated *B. edulis*, a higher 1-octen-3-ol and (E)-2-octen-1-ol content was found in ultrasonicated samples (9.0% and 17.9% higher, respectively), and no 3-octanol was established in ultrasonicated *B. edulis*. Comparing the VC in thermally treated and ultrasonicated unfermented *C. cibarius* samples, the content of the main VC (1-octen-3-ol and oct-(2E)-enal) remained similar, but the hexanal content in thermally treated *C. cibarius* was 1.5 times higher than that in ultrasonicated samples. The content of the main VC in *R. caperata* (benzaldehyde and 1-octen-3-ol) was similar in thermally treated and ultrasonicated samples (on average, 48.5% and 33.8%, respectively, of the total VC content).

Usually, the high temperature elicits a significant decrease in total volatile compounds because most of these compounds are thermosensitive [43]. Selli et al. [44] reported that boiling and oven-cooking significantly reduced the content of VC in the raw champignon and oyster mushrooms, while the main key odourants of tested mushrooms were 1-octen-3-one, methional, and 1-octen-3-ol. However, Politowicz et al. [43] found that the higher drying temperature increased the total concentrations of volatile compounds in oyster mushrooms. Pérez-Santaescolástica et al. [45] reported that low-temperature treatment and its combination with ultrasound significantly decreased the total volatile content in dry-cured hams.

The greatest variety of VC was shown in thermally treated and/or ultrasonicated fermented samples (Table 4). In most of the fermented mushroom samples, 1-octen-3-ol was the main VC (except in thermally treated and fermented *C. cibarius* in which the main VC was oct-(2E)-enal). Considering the influence of the treatment before fermentation on the 1-octen-3-ol content in mushrooms, various tendencies were established: in thermally treated and fermented *B. edulis*, the 1-octen-3-ol content was, on average, 2.0 times higher than in ultrasonicated fermented samples; however, in *C. cibarius* and *R. caperata*, a higher 1-octen-3-ol content was established in ultrasonicated and fermented samples (on average, 1.6 and 1.5 times higher, respectively). Other VC which accounted for more than 5% of the VC profile were: (E)-2-octen-1-ol in thermally treated and fermented *B. edulis*; 3-methyl-1-butanol, 3-octanone, and 3-octanol in ultrasonicated and fermented *B. edulis*; acetoin, 3-methyl-1-butanol, 3-octanone, and 2-pentylfuran in thermally treated and fermented *C. cibarius*; 3-octanone, 3-octanol, oct-(2E)-enal, and 1-octanol in ultrasonicated and fermented *C. cibarius*; hexanal, 2-heptenal, and oct-(2E)-enal in thermally treated and fermented *R. caperata*; and 3-methyl-1-butanol, ethyl tiglate, benzaldehyde, 3-octanone, and hexanoic acid ethyl ester in ultrasonicated and fermented *R. caperata*.

In most of the fermented mushroom samples, 1-octen-3-ol was the main VC (except in thermally treated and fermented *C. cibarius*, in which the main VC was oct-(2E)-enal). Considering the influence of the treatment before fermentation on the 1-octen-3-ol content in mushrooms, various tendencies were established: in thermally treated and fermented *B. edulis*, the 1-octen-3-ol content was, on average, 2.0 times higher than in ultrasonicated fermented samples; however, in *C. cibarius* and *R. caperata*, a higher 1-octen-3-ol content was established in ultrasonicated and fermented samples (on average, 1.6 and 1.5 times higher, respectively). Other VC which accounted for more than 5% of the VC profile were: (E)-2-octen-1-ol in thermally treated and fermented *B. edulis*; 3-methyl-1-butanol, 3-octanone, and 3-octanol in ultrasonicated and fermented *B. edulis*; acetoin, 3-methyl-1-butanol, 3-octanone, and 2-pentylfuran in thermally treated and fermented *C. cibarius*; 3-octanone, 3-octanol, oct-(2E)-enal, and 1-octanol in ultrasonicated and fermented *Cantharellus*; hexanal, 2-heptenal, and oct-(2E)-enal in thermally treated and fermented *R. caperata*; and 3-methyl-1-butanol, ethyl tiglate, benzaldehyde, 3-octanone, and hexanoic acid ethyl ester in ultrasonicated and fermented *R. caperata*.

The odour of benzaldehyde is described as almond, fruity, powdery, nutty, and benzaldehyde-like, and the taste is sweet, oily, almond, cherry, nutty, and woody; the odour of hexanal is green, fatty, leafy, vegetative, fruity, and clean with a woody nuance, and the taste is green, woody, vegetative, apple, grassy, citrus, and orange with a fresh, lingering aftertaste; the odour of 3-methyl-1-butanol is fusel, alcoholic, pungent, ethereal, cognac, fruity, banana, and molasses, and the taste is fusel, fermented, fruity, banana, ethereal, and cognac; 3-octanone, used as a flavour and fragrance ingredient [46,47] and can be found in pine king bolete (*B. pinophilus*) [46]; its odour is described as earthy, mushroom, dairy, musty, creamy, and waxy with a slight fermented green minty nuance, and its taste is musty, mushroom, earthy, and creamy dairy. Acetoin has a buttery-type odour, and in more detail is described as sweet, buttery, creamy, dairy, milky, and fatty; the odour of 2-pentylfuran is described as fruity, green, earthy, and beany with vegetable-like nuances, and the taste as green, waxy, with musty and cooked caramel nuances; the odour of 2-heptenal is sweet, fruity, tutti frutti, tropical, berry, floral, and caramel; that of ethyl tiglate is sweet, fruity, pineapple, waxy, fatty, and estery with a green banana nuance [41].

In the present study, the increased variety of VC in fermented mushrooms is related to the fact that most LAB strains, as well as *Lactobacillus*, produce unique flavour profiles in fermented foods [48]. VC production can be performed through proteolysis, lipolysis, carbohydrate, and fatty acid metabolism, as well as amino acid catabolism [49]. Biochemical constituents of foods are hydrolysed by LAB enzymes into flavour compounds and further transformed into VC. The profile of these compounds strongly depends on the LAB strain, substrate availability, salt stress, and technological factors such as pH, temperature, and water activity [50].

### 3.4. Overall Acceptability and Emotions Induced in Consumers by the Edible Mushrooms

The overall acceptability and emotions induced in consumers by edible mushrooms are given in Table 5. No significant differences in overall acceptability were found. This could be explained by both unfermented and fermented edible mushrooms being a traditional food in Lithuania. Similarly, no correlations were established between the pH of the samples and their overall acceptability.

However, significant differences were found between the emotions induced in judges. The emotion ‘neutral’ was expressed most strongly for the thermally treated unfermented samples of all species and for ultrasonicated and fermented *B. edulis* samples (on average, 0.8086), and a moderate positive correlation was found between the emotion ‘neutral’ and samples’ pH (r = 0.5720, *p* = 0.0001). The emotion ‘happy’ was expressed most strongly for ultrasonicated and fermented *B. edulis* samples. Other samples induced significantly weaker expression of the emotion ‘happy’, and a moderate positive correlation was found between the emotion ‘happy’ and samples’ yellowness (b*) (r = 0.4410, *p* = 0.007). Ultrasonicated and fermented *B. edulis* samples also induced the highest intensity of the emotions ‘sad’, ‘angry’, ‘surprised’, and ‘contempt’, and a moderate positive correlation was found between the emotion ‘angry’ and samples’ pH (r = 0.7920, *p* = 0.0001). The strongest expression of the emotion ‘scared’ was established for thermally treated *R. caperata* samples. This perhaps could be explained by these mushrooms looking similar to toxic ones.

Correlations between the VC and overall acceptability of thermally treated and ultrasonicated unfermented and fermented mushrooms, as well as between VC and the emotion ‘happy’, are given in Supplementary file Tables S2 and S3, respectively. No significant correlations were established between the VC and overall acceptability of thermally treated and ultrasonicated unfermented mushrooms (except for a moderate positive correlation between the overall acceptability of the fermented mushrooms and their 3-octanone content; r = 0.519, *p* = 0.027); however, significant correlations were found between the emotion ‘happy’ and hexanal, benzaldehyde, 3-octanone, octanal, 3-ethyl-2-methyl-1,3-hexadiene, oct-(2E)-enal, 6-methyl-hept-2-en-4-ol, and nonanal (r = 0.619, *p* = 0.006; r = −0.472, *p* = 0.048; r = −0.472, *p* = 0.048; r = −0.862, *p* = 0.0001; r = 0.842, *p* = 0.0001; r = 0.778, *p* = 0.0001; r = 0.628, *p* = 0.005; r = −0.530, *p* = 0.024, respectively). It can be concluded that an increase in the content of benzaldehyde, 3-octanone, octanal, and nonanal reduced the intensity of the ‘happy’ emotion expression in consumers. In contrast, increasing the content of hexanal, 3-ethyl-2-methyl-1,3-hexadiene, oct-(2E)-enal, and 6-methyl-hept-2-en-4-ol led to the stronger expression of the emotion ‘happy’.

Significant positive correlations were also established between the emotion ‘happy’ induced by fermented mushrooms and 3-methyl-1-butanol, 2,3-butanediol, 3-octanone, 3-octanol, 2-iodo-3-methyl-butane, and N-hexyl-1-hexanamine (r = 0.755, *p* = 0.0001; r = 0.999, *p* = 0.0001; r = 0.819, *p* = 0.0001; r = 0.622, *p* = 0.006; r = 0.999, *p* = 0.0001; r = 0.999, *p* = 0.0001, respectively). An increase in the percentage of benzaldehyde, oct-(2E)-enal, (E)-2-octen-1-ol, and 2-nonanone in the VC profile of fermented mushrooms reduced the intensity of the ‘happy’ emotion expression (r = −0.563, *p* = 0.0023; r = −0.518, *p* = 0.0028; r = −0.564, *p* = 0.0015; r = −0.617, *p* = 0.006, respectively).

Our study showed that the liking scores of treated mushrooms were similar. Contrary to our results, in the study of Aisala et al. [40], consumer clusters with significantly different likings of wild Nordic mushrooms were found. Zhang et al. [51] reported that the high hedonic liking of porcini mushrooms was related to 3-(methylthio)propanal, 3-(methylthio)propanol, pyrazines, phenols, and furanone, whereas the low hedonic liking correlated with high levels of 1-octen-3-ol, octanal, 2-pentylfuran, and 3-methylbutanoic acid. Sensory acceptability is commonly used to evaluate liking for foods. However, in recent years, most studies have included emotions in food assessments alongside overall liking [52]. Similar to our study, Yang et al. [53] reported that the sensory analysis of products made with Bambara flour showed no significant differences between tested categories, while differences were observed in emotional response. This indicates that emotional response could be a more sensitive approach to measuring consumer perception of products. To the best of our knowledge, studies on mushroom-evoked emotional responses are very scarce. Only Tepsongkroh et al. [54] reported that the mushroom additive in extruded snack formulations significantly influenced the sensory liking and emotional profile, measured with the Essense Profile^®^, of these products.

### 3.5. BA Content in Edible Mushrooms

**Figure 5:**
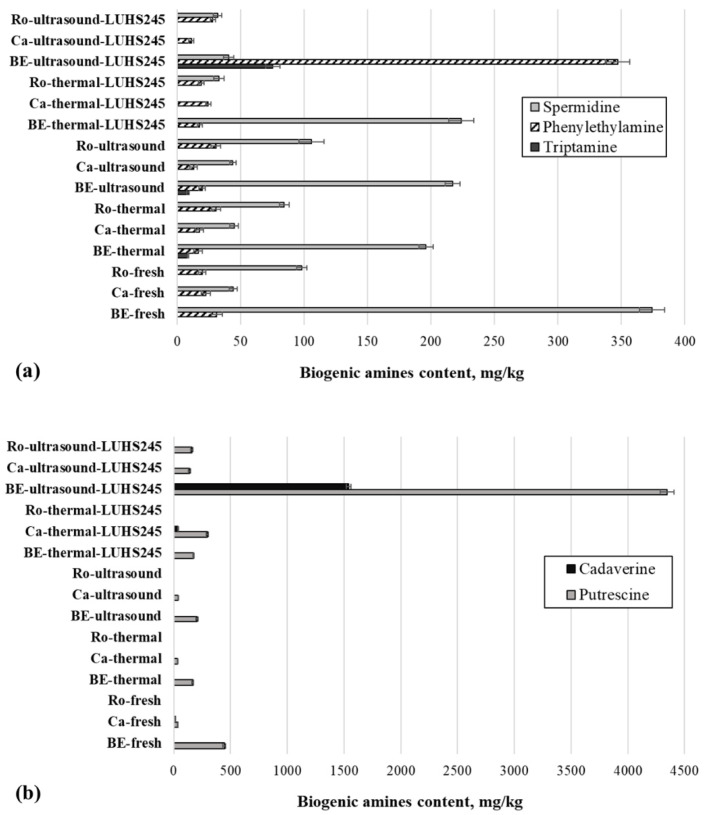

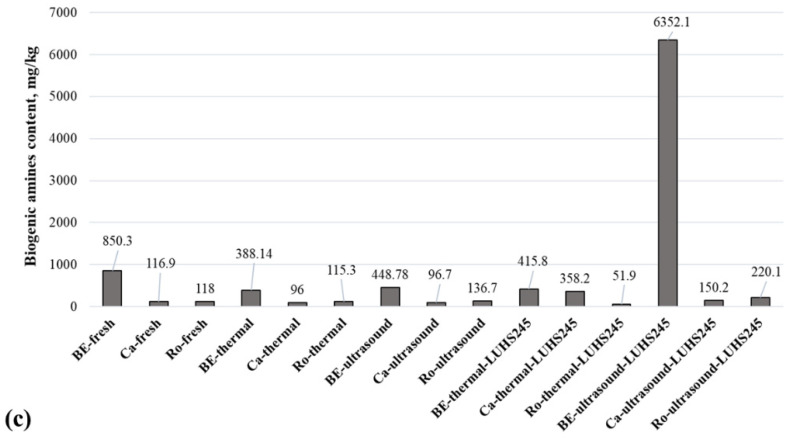
(**a**) The content of spermidine, phenylethylamine, and triptamine in edible mushrooms; (**b**) the content of cadaverine and putrescine in edible mushrooms; and (**c**) the sum of biogenic amines in edible mushrooms (BE—*Boletus edulis*; Ca—*Cantharellus* *cibarius*; Ro—*Rozites caperata*; thermal—thermally treated by boiling for 30 min; ultrasound—ultrasonicated for 30 min; LUHS245—fermented with *L. uvarum* LUHS245).

The content of separate BA (Figure 5a,b) and their sum (Figure 5c) in mushroom samples is shown in Figure 5. Histamine, tyramine, and spermine were not found in all samples. Phenylethylamine and spermidine were found in all fresh mushroom samples, the highest concentrations being found in *B. edulis* (31.3 and 374 mg/kg, respectively). Putrescine was found in *B. edulis* and *C. cibarius* (445 and 35.2 mg/kg, respectively). In addition, cadaverine was established in fresh *C. cibarius* (14.6 mg/kg). Comparing the sum of BA in fresh mushrooms, the highest was established *B. edulis*; that in *C. cibarius* and *R. caperata* was 7.3 and 7.2 times lower, respectively.

In comparing thermally treated and ultrasonicated unfermented mushroom groups, tryptamine was found in thermally treated and ultrasonicated *B. edulis* (8.54 and 8.88 mg/kg, respectively). As in fresh samples, phenylethylamine and spermidine were established in all thermally treated and ultrasonicated mushrooms. Additionally, putrescine was found in both thermally treated and ultrasonicated *B. edulis* and *C. cibarius*. However, the sum of BA in thermally treated and ultrasonicated samples was lower in treated samples of *B. edulis* and *C. cibarius* than in fresh mushrooms: 2.2 and 1.2 times lower, respectively, in thermally treated samples, and 1.9 and 1.2 times lower, respectively, in ultrasonicated samples.

When comparing thermally treated and ultrasonicated fermented mushroom groups, tryptamine was found in ultrasonicated and fermented *B. edulis* (75.5 mg/kg), and cadaverine was established in both thermally treated and ultrasonicated fermented *B. edulis* samples (38.6 and 1542 mg/kg, respectively). Ultrasonication before fermentation, in comparison with thermal treatment before fermentation, increased the sum of BA in *B. edulis* and *R. caperata* samples (on average, by 15.3 and 2.4 times, respectively). In contrast, ultrasonication before fermentation reduced the sum of BA in *C. cibarius* samples (on average, by 2.4 times).

Microbial activities could lead to amino acid decarboxylation which induces BA formation. [55]. The amount and type of BA in food are closely related to the spoilage and fermentation processes and depend on the composition of food (mainly free amino acid content), microorganisms in it, and technological and environmental conditions [56]. Acidity parameters (especially pH) are also important factors for the formation of BA [57]. However, no correlation was found between the BA content and the pH of samples in our study. A moderate positive correlation was established between spermidine and the mould/yeast count in samples (r = 0.585, *p* ≤ 0.0001). This could be explained by the fact that moulds and yeasts could also contribute to BA production [14]. However, it should be noted that the metabolic pathways available in LAB generate significant concentrations of BA in fermented foods [15]. Certain BA, such as histamine, tyramine, cadaverine, and putrescine, can be produced by LAB species in different fermented foods [14]. The routes available for this are the enzymatic production of putrescine from ornithine and/or from arginine via agmatine, followed by the transformation of agmatine to N-carbamoylputrescine, and then on to putrescine, which is converted to spermine and then to spermidine; cadaverine from lysine; 2-phenylethylamine from phenylalanine; tyramine from tyrosine; histamine from histidine; tryptamine from tryptophan; and trimethylamine from trimethylamine-N-oxide [16]. The reduced level of BA in treated samples could be explained by the inactivation of decarboxylase positive microorganisms by the thermal treatment or ultrasonication of raw material. High-intensity ultrasound not only eliminates microorganisms and/or denatures enzymes, but also facilitates the extraction processes [33], which may result in the greater release of accumulated BA from the food material matrix. It was reported that thermal processing reduces spermine and spermidine; however, these BA are very difficult to destroy by processing [58].

The total BA content in food should not exceed 750–900 mg/kg [59]. According to the obtained results in the present study, all tested samples, except ultrasonicated and fermented *B. edulis*, were in this range. Toxicological effects and various health disorders (cardiovascular, gastrointestinal, respiratory, and neurological) are related to the high concentration of BA consumed by humans [60]. These effects are mainly associated with tyramine and histamine, but other BA such as putrescine, cadaverine, and phenylethylamine could strengthen the toxic effect of histamine [56]. However, in our study, histamine and tyramine were not found in all samples. 

Studies on BA in mushrooms are quite scarce and focus on raw or processed (boiled, dried) mushrooms, but the reported concentrations of BA significantly differ among species [58,61,62]. Similar to our study, Jabłońska-Ryś et al. [61] found that spermidine and putrescine were the most common BA in processed and unprocessed mushrooms available in the Polish market. Reis et al. [62] reported the presence of spermidine, phenylethylamine, tyramine, and tryptamine in fresh edible commercial mushroom species. To date, to the best of our knowledge, fermented *B. edulis*, *C. cibarius*, *R. caperata,* or even other fermented mushrooms have not been analysed for the content of BA. 

## 4. Conclusions

Although lactic acid fermentation is traditionally used to preserve mushrooms, studies in this field are still scarce. Therefore, there is a need to analyse the impact of this biological approach on the quality and safety of mushrooms. In the present study, the quality and safety traits of edible mushrooms fermented with *L. casei* LUHS210 and *L. uvarum* LUHS245 were evaluated. As pre-treatment, ultrasonication or prolonged thermal treatment were applied before fermentation. Pre-treatment significantly affected physicochemical properties, while the greatest variety of volatile compounds was obtained after pre-treatment and fermentation of tested mushrooms. The emotional response was a more sensitive approach to measuring the perception of the tested mushrooms. All ultrasonicated and fermented *B. edulis* samples elicited the expression ‘happy’. The lowest sum of BA was found in thermally pre-treated and fermented *R. caperata,* while the highest one was in ultrasonicated and fermented *B. edulis*. Finally, it can be stated that despite good overall acceptability, it is important to select appropriate LAB strains for the fermentation of edible mushrooms to ensure their safety in the case of BA formation.

## Figures and Tables

**Table 1:** Colour coordinates (L* lightness; a* redness or -a* greenness; b* yellowness or -b* blueness); and pH of the edible mushrooms.

Edible Mushroom Samples	Colour Coordinates, NBS			pH	Mould and Yeast Count, log_10_ CFU/g	
	L*	a*	b*		BE-Fresh	3.63 ± 0.28 ^A^
					Ca-Fresh	2.98 ± 0.31 ^A^
					Ro-Fresh	3.13 ± 0.24 ^A^
	Thermal Treated Non-Fermented					
BE-thermal	29.19 ± 0.22 ^a,A^	5.17 ± 0.21 ^b,C^	5.21 ± 0.10 ^b,B^	5.92 ± 0.26 ^a,F^	3.02 ± 0.21 ^a,C^	
Ca-thermal	43.25 ± 0.14 ^a,F^	7.19 ± 0.11 ^a,E^	21.76 ± 0.19 ^a,D^	7.11 ± 0.17 ^a,G^	2.55 ± 0.24 ^a,C,B^	
Ro-thermal	42.30 ± 0.19 ^a,E^	5.71 ± 0.09 ^a,D^	20.99 ± 0.18 ^a,C^	5.72 ± 0.15 ^a,E,F^	2.42 ± 0.18 ^b,C,B^	
	Ultrasonicated non-fermented					
BE-ultrasound	29.76 ± 0.21 ^b,B^	4.45 ± 0.11 ^a,B^	4.75 ± 0.05 ^a,A^	5.90 ± 0.04 ^a,F^	3.01 ± 0.15 ^a,C^	
Ca-ultrasound	43.10 ± 0.19 ^a,F^	15.61 ± 0.27 ^b,H^	31.34 ± 0.13 ^b,H^	7.15 ± 0.08 ^a,G^	2.83 ± 0.26 ^a,C^	
Ro-ultrasound	51.23 ± 0.24 ^b,H^	9.62 ± 0.10 ^b,F^	29.70 ± 0.21 ^b,G^	5.71 ± 0.04 ^a,E^	2.04 ± 0.19 ^a,B^	
	Thermal treated and fermented with *L. uvarum* LUHS245 strain					
BE-thermal-_LUHS245_	32.14 ± 0.23 ^a,C^	5.19 ± 0.14 ^a,C^	5.05 ± 0.09 ^a,B^	3.97 ± 0.04 ^a,C^	2.46 ± 0.22 ^a,C,B^	
Ca-thermal-_LUHS245_	51.98 ± 0.27 ^b,I^	14.66 ± 0.11 ^a,G^	38.07 ± 0.21 ^a,I^	4.09 ± 0.03 ^a,D^	2.13 ± 0.20 ^a,B^	
Ro-thermal-_LUHS245_	56.59 ± 0.18 ^a,J^	4.27 ± 0.08 ^a,A^	23.67 ± 0.07 ^a,E^	3.41 ± 0.05 ^a,B^	1.97 ± 0.17 ^a,B^	
	Ultrasonicated and fermented with *L. uvarum* LUHS245 strain					
BE-ultrasound-_LUHS245_	33.18 ± 0.22 ^b,D^	5.29 ± 0.08 ^a,C^	5.10 ± 0.04 ^a,B^	3.96 ± 0.05 ^a,C,D^	2.17 ± 0.21 ^a,B^	
Ca-ultrasound-_LUHS245_	50.07 ± 0.15 ^a,G^	16.52 ± 0.11 ^b,I^	39.82 ± 0.18 ^b,J^	4.11 ± 0.06 ^a,D^	2.21 ± 0.23 ^a,B^	
Ro-ultrasound-_LUHS245_	60.98 ± 0.18 ^b,K^	4.46 ± 0.06 ^b,B^	26.95 ± 0.21 ^b,F^	3.55 ± 0.04 ^b,A^	2.12 ± 0.13 ^a,B^	

BE—*Boletus edulis*; Ca—*Cantharellus cibarius*; Ro—*Rozites caperata*; thermal—thermal treated by boiling 30 min; ultrasound—ultrasonicated for 30 min; LUHS245—fermented with *L. uvarum* LUHS245 strain. L* lightness; a* redness or -a* greenness; b* yellowness or -b* blueness; NBS—National Bureau of Standards units. Data are represented as means (n = 3, replicates of analysis) ± SD; ^a–b^ for the same analytical parameters, between thermal treated and ultrasonicated (non-fermented and fermented groups were compared separately), and same species edible mushroom groups; means with different letters are significantly different (*p* ≤ 0.05); ^A–K^ for the same analytical parameters, in all edible mushroom samples; means with different letters are significantly different (*p* ≤ 0.05).

**Table 2:** Volatile compounds (in whole profile >1%) of non-treated mushrooms (% from the total volatile compounds).

RT, Min	VC	BE—Non-Treated	Ca—Non-Treated	Ro—Non-Treated
5.763	Hexanal	nd	1.89 ± 0.17 ^b^	0.445 ± 0.025 ^a^
9.949	Benzaldehyde	0.538 ± 0.027 ^a^	1.27 ± 0.13 ^b^	35.6 ± 2.5 ^c^
10.493	1-Octen-3-ol	61.9 ± 2.5 ^b^	55.2 ± 0.51 ^a^	50.4 ± 3.8 ^a^
10.669	3-Octanone	5.62 ± 0.34 ^b^	nd	1.81 ± 1.6 ^a^
10.885	3-Octanol	8.81 ± 0.72 ^c^	0.525 ± 0.049 ^a^	3.71 ± 2.7 ^b^
11.091	Octanal	5.47 ± 0.61 ^c^	0.367 ± 0.028 ^a^	1.34 ± 0.21 ^b^
11.844	3-ethyl-2-methyl-1,3-hexadiene	nd	1.82 ± 0.26	nd
12.476	Oct-(2E)-enal	4.55 ± 0.39 ^b^	16.3 ± 0.58 ^c^	2.25 ± 0.23 ^a^
12.728	(E)-2-octen-1-ol	nd	16.9 ± 0.18 ^b^	1.13 ± 0.11 ^a^
12.788	1-Octanol	8.01 ± 0.78	nd	nd

RT—retention time; BE—*Boletus edulis*; Ca—*Cantharellus cibarius*; Ro—*Rozites caperata;* VC—volatile compound; nd—not detected. Data are represented as means (n = 3, replicates of analysis) ± SD; ^a–c^ for the same analytical parameters between mushrooms; means with different letters are significantly different (*p* ≤ 0.05).

**Table 3:** Volatile compounds (in whole profile >1%) of thermal treated and ultrasonicated non-fermented mushrooms (% from the total volatile compounds).

RT, Min	VC	BE—Thermal	Ca—Thermal	Ro—Thermal	BE—Ultrasound	Ca—Ultrasound	Ro—Ultrasound
5.763	Hexanal	0.922 ± 0.054 ^B^	5.32 ± 0.49 ^b,C^	0.986 ± 0.074 ^b,B^	nd	3.46 ± 0.31 ^a^	0.618 ± 0.059 ^a,A^
8.11	2-Heptanone	1.05 ± 0.09	nd	nd	nd	nd	nd
8.424	Heptanal	2.06 ± 0.18 ^a,B^	0.357 ± 0.031 ^a,A^	nd	1.90 ± 0.18 ^a,B^	0.323 ± 0.029 ^a,A^	nd
9.949	Benzaldehyde	4.53 ± 0.36 ^b,C^	1.97 ± 0.17 ^a,A^	44.5 ± 3.9 ^a,D^	1.53 ± 0.16 ^a,A^	2.22 ± 0.23 ^a,B^	51.6 ± 4.4 ^a,D^
10.493	1-Octen-3-ol	57.9 ± 3.8 ^a,C^	51.1 ± 4.2 ^a,B^	35.9 ± 2.8 ^a,A^	63.6 ± 0.59 ^b,C^	50.9 ± 4.6 ^a,B^	31.7 ± 2.9 ^a,A^
10.669	3-Octanone	nd	nd	4.57 ± 0.48 ^a,A^	nd	nd	4.35 ± 0.38 ^a,A^
10.885	3-Octanol	7.00 ± 0.69 ^D^	1.29 ± 0.13 ^b,B^	6.08 ± 0.57 ^b,D^	nd	0.602 ± 0.058 ^a,A^	4.20 ± 0.31 ^a,C^
11.091	Octanal	1.27 ± 0.11 ^a,C^	0.651 ± 0.045 ^b,B^	1.51 ± 0.14 ^c,C,D^	1.35 ± 0.14 ^a,C^	0.436 ± 0.041 ^a,A^	1.49 ± 0.13 ^c,C^
11.673	p-Cymene	0.955 ± 0.043 ^a,A^	nd	nd	1.48 ± 0.13^b,B^	nd	nd
11.788	Limonene	3.55 ± 0.28 ^a,B^	nd	nd	4.60 ± 0.36 ^b,C^	nd	0.409 ± 0.032 ^A^
11.844	3-ethyl-2-methyl-1,3-hexadiene	nd	2.80 ± 0.27 ^a,A^	nd	nd	3.10 ± 0.028 ^b,B^	nd
12.476	Oct-(2E)-enal	3.16 ± 0.29 ^a,B^	22.2 ± 1.3 ^a,D^	2.21 ± 0.23 ^a,A^	4.45 ± 0.42 ^b,C^	20.7 ± 1.8 ^a,D^	2.50 ± 0.23 ^a,A^
12.728	(E)-2-octen-1-ol	9.85 ± 0.41 ^a,D^	4.54 ± 0.45 ^a,C^	0.519 ± 0.049 ^b,B^	12.0 ± 0.13 ^b,E^	11.28 ± 1.3 ^b,E^	0.213 ± 0.019 ^a,A^
13.341	6-Methyl-hept-2-en-4-ol	nd	1.40 ± 0.15^b,B^	nd	nd	0.877 ± 0.065 ^a,A^	nd
13.612	Nonanal	1.16 ± 0.12 ^a,C^	0.907 ± 0.073 ^b,B^	0.962 ± 0.081 ^b,B^	1.48 ± 0.15 ^a,C^	0.641 ± 0.047 ^a,A^	0.718 ± 0.053 ^a,A^
15.611	3,6-Dimethyl-2,3,3a,4,5,7a-hexahydrobenzofuran	1.41 ± 0.13 ^a,A^	nd	nd	1.53 ± 0.16 ^a,A^	nd	nd
16.731	Heptylidene acetone	0.663 ± 0.059 ^a,A^	nd	nd	1.07 ± 0.09 ^b,B^	nd	nd

RT—retention time; BE—*Boletus edulis*; Ca—*Cantharellus cibarius*; Ro—*Rozites caperata;* VC—volatile compound; thermal—thermal treated by boiling 30 min; ultrasound—ultrasonicated for 30 min; nd—not detected. Data are represented as means (n = 3, replicates of analysis) ± SD; ^a–b^ for the same analytical parameters between thermal treated and ultrasonicated the same species edible mushroom groups; means with different letters are significantly different (*p* ≤ 0.05); ^A–E^ for the same analytical parameters in all edible mushroom samples; means with different letters are significantly different (*p* ≤ 0.05).

**Table 4:** Volatile compound profile of thermal treated and ultrasonicated fermented edible mushrooms.

RT, Min	VC	BE—Thermal-LUHS245	Ca—Thermal-LUHS245	Ro—Thermal-LUHS245	BE—Ultrasound-LUHS245	Ca—Ultrasound-LUHS245	Ro—Ultrasound-LUHS245
2.375	Acetic acid	nd	nd	2.32 ± 0.22	nd	nd	nd
3.779	Acetoin	nd	14.8 ± 0.13 ^B^	2.68 ± 0.24 ^A^	2.76 ± 0.26 ^A^	nd	nd
4.355	3-methyl-1-butanol	nd	13.0 ± 0.58 ^b,B^	nd	18.1 ± 0.52 ^C^	1.86 ± 0.19 ^a,A^	6.98 ± 0.65
5.277	2,3-Butanediol	nd	nd	nd	3.73 ± 0.34	nd	nd
5.763	Hexanal	nd	nd	16.8 ± 0.17 ^b,C^	nd	3.16 ± 0.32 ^B^	1.69 ± 0.18 ^a,A^
7.164	3-methyl-butanoic acid ethyl ester	nd	nd	nd	nd	nd	2.12 ± 0.23
7.567	1-Hexanol	nd	nd	1.09 ± 0.10 ^a,A^	nd	2.72 ± 0.25 ^B^	5.08 ± 0.41 ^b,C^
8.11	2-Heptanone	1.83 ± 0.16 ^B^	nd	nd	nd	nd	0.312 ± 0.24 ^A^
9.451	Ethyl tiglate	nd	3.31 ± 0.27 ^A^	nd	2.90 ± 0.28 ^A^	nd	14.1 ± 0.83 ^B^
9.631	2,7-dimethyl-4,5-octanediol	nd	1.46 ± 0.12	nd	nd	nd	nd
9.865	2-Heptenal	nd	0.545 ± 0.041 ^b,B^	9.02 ± 0.72 ^C^	nd	0.456 ± 0.041 ^a,A^	nd
9.949	Benzaldehyde	4.67 ± 0.39 ^B^	1.65 ± 0.15 ^a,A^	nd	nd	1.75 ± 0.16 ^a,A^	6.32 ± 0.59 ^C^
10.244	Heptyl formate	nd	nd	nd	nd	1.11 ± 0.12	nd
10.493	1-Octen-3-ol	52.7 ± 0.48 ^b,F^	22.2 ± 1.3 ^a,C^	7.30 ± 0.59 ^a,A^	26.8 ± 1.3 ^a,D^	36.5 ± 2.8 ^b,E^	11.1 ± 0.12 ^b,B^
10.584	2,5-Octanedione	nd	nd	3.39 ± 0.32	nd	nd	nd
10.669	3-Octanone	nd	7.32 ± 0.56 ^a,A^	nd	26.4 ± 2.1 ^C^	12.8 ± 1.1 ^b,B^	12.5 ± 0.9 ^B^
10.812	2-pentylfuran	nd	7.09 ± 0.48 ^B^	3.53 ± 0.32 ^A^	nd	nd	nd
10.885	3-Octanol	nd	nd	3.26 ± 0.30 ^a,A^	9.12 ± 0.83 ^C^	7.46 ± 0.62 ^C^	5.46 ± 0.42 ^b,B^
10.988	Hexanoic acid ethyl ester	nd	nd	nd	nd	nd	5.24 ± 0.48
11.091	Octanal	1.50 ± 0.14 ^A^	nd	2.82 ± 0.25 ^B^	nd	1.72 ± 0.16 ^A^	nd
11.788	Limonene	3.64 ± 0.32 ^b,C^	0.587 ± 0.051 ^B^	nd	0.151 ± 0.11 ^a,A^	nd	0.188 ± 0.015 ^A^
11.844	3-ethyl-2-methyl-1,3-hexadiene	nd	nd	2.03 ± 0.18 ^A^	nd	3.32 ± 0.29 ^B^	nd
11.883	Benzyl alcohol	nd	nd	nd	nd	nd	3.11 ± 0.31
12.476	Oct-(2E)-enal	4.32 ± 0.41 ^b,D^	1.52 ± 0.14 ^a,B^	9.94 ± 0.85 ^b,F^	0.320 ± 0.28 ^a,A^	7.66 ± 0.59 ^b,E^	2.06 ± 0.19 ^a,C^
12.728	(E)-2-octen-1-ol	9.62 ± 0.83 ^D^	3.58 ± 0.31 ^b,B^	4.23 ± 0.38 ^b,C^	nd	1.98 ± 0.17 ^a,A^	3.00 ± 0.28 ^a,B^
12.788	1-Octanol	nd	nd	nd	2.53 ± 0.41^A^	11.6 ± 0.12^B^	nd
13.267	2-iodo-3-methyl-butane	nd	nd	nd	1.07 ± 0.10	nd	nd
13.314	2-Nonanone	4.14 ± 0.25 ^A^	4.97 ± 0.41 ^A^	4.16 ± 0.36 ^a,A^	nd	nd	4.98 ± 0.43 ^a,A^
13.612	Nonanal	1.44 ± 0.12 ^D^	1.10 ± 0.10 ^b,C^	4.39 ± 0.41 ^b,E^	nd	0.642 ± 0.059 ^a,A^	0.918 ± 0.058 ^a,B^
13.865	Phenylethyl Alcohol	nd	1.44 ± 0.13 ^b,C^	0.593 ± 0.042 ^a,A^	1.80 ± 0.17 ^D^	0.803 ± 0.065 ^a,B^	2.54 ± 0.25 ^b,E^
13.945	N-hexyl-1-hexanamine	nd	nd	nd	1.64 ± 0.15	nd	nd
14.922	(E)-non-2-enal	0.327 ± 0.029 ^B^	0.571 ± 0.041 ^a,C^	1.96 ± 0.18 ^b,E^	nd	0.844 ± 0.079 ^b,D^	0.252 ± 0.021 ^a,A^
15.611	3,6-Dimethyl-2,3,3a,4,5,7a-hexahydrobenzofuran	1.04 ± 0.09	nd	nd	nd	nd	nd
15.753	Octanoic acid ethyl ester	nd	0.560 ± 0.052 ^B^	nd	0.154 ± 0.014 ^A^	nd	1.34 ± 0.11 ^C^
16.171	(E,E)-2,4-nonadienal	0.129 ± 0.11 ^b,B^	nd	2.27 ± 0.21 ^b,E^	0.029 ± 0.003 ^a,A^	0.179 ± 0.015 ^C^	0.248 ± 0.023 ^a,D^
16.416	2,6,6-trimethyl-1-cyclohexene-1-carboxaldehyde	nd	1.26 ± 0.13 ^b,B^	nd	nd	0.370 ± 0.029 ^a,A^	nd
17.206	Dec-(2E)-enal	nd	nd	1.66 ± 0.15 ^b,B^	nd	nd	0.276 ± 0.026 ^a,A^
17.293	Nonanoic acid	2.37 ± 0.22 ^b,D^	3.41 ± 0.28 ^b,E^	1.96 ± 0.17 ^B^	0.170 ± 0.014 ^a,A^	0.300 ± 0.025 ^a,C^	nd
18.376	Deca-(2E,4E)-dienal	nd	nd	1.13 ± 0.10 ^b,D^	0.020 ± 0.03 ^A^	0.215 ± 0.019 ^B^	0.287 ± 0.027 ^a,C^
19.656	trans-4,5-Epoxy-(E)-2-decenal	nd	nd	1.33 ± 0.12	nd	nd	nd
21.48	9-Decen-1-yl acetate	1.28 ± 0.13 ^C^	0.209 ± 0.019 ^A^	0.596 ± 0.046 ^B^	nd	nd	nd
21.88	4-(2,6,6-trimethyl-1-cyclohexen-1-yl)-3-buten-2-one	nd	2.29 ± 0.23 ^b,B^	nd	nd	0.725 ± 0.047 ^a,A^	nd
23.051	Dodecanoic acid	nd	nd	1.65 ± 0.14 ^B^	0.036 ± 0.004 ^A^	nd	nd

RT—retention time; BE—*Boletus edulis*; Ca—*Cantharellus cibarius*; Ro—*Rozites caperata;* VC—volatile compound; LUHS245—fermented with *L. uvarum* LUHS245 strain; thermal—thermal treated by boiling 30 min; ultrasound—ultrasonicated for 30 min; nd—not detected. Data are represented as means (n = 3, replicates of analysis) ± SD; ^a–b^ for the same analytical parameters between thermal treated and ultrasonicated fermented edible mushroom groups; means with different letters are significantly different (*p* ≤ 0.05); ^A–F^ for the same analytical parameters in all edible mushroom samples; means with different letters are significantly different (*p* ≤ 0.05).

**Table 5:** Overall acceptability and emotions induced in consumers by edible mushrooms.

Edible Mushroom Samples	OA	Emotions Induced by the Samples (from 0 to 1)								
		Neutral	Happy	Sad	Angry	Surprised	Scared	Disgusted	Contempt	Valence
	**Thermal treated non-fermented**									
BE-thermal	7.6 ± 1.2 ^a^	0.8068 ± 0.0096 ^d^	0.0106 ± 0.0015 ^d^	0.0675 ± 0.0028 ^c^	0.0224 ± 0.0018 ^c^	0.0086 ± 0.0017 ^d^	0.0037 ± 0.0006 ^d^	0.0156 ± 0.0016 ^a^	0.0014 ± 0.0004 ^c^	0.0852 ± 0.0075 ^b^
Ca-thermal	8.9 ± 2.3 ^a^	0.8010 ± 0.0048 ^d^	0.0177 ± 0.0019 ^c^	0.0135 ± 0.0011^i^	0.0322 ± 0.0025 ^b^	0.0059 ± 0.0006 ^e^	0.0014 ± 0.0005 ^e^	0.0034 ± 0.0005 ^e^	0.0011 ± 0.0003 ^c^	0.0269 ± 0.0034 ^f^
Ro-thermal	7.6 ± 1.4 ^a^	0.8196 ± 0.0115 ^d^	0.0015 ± 0.0006 ^f^	0.0433 ± 0.0021 ^e^	0.0263 ± 0.0023 ^c^	0.0024 ± 0.0004 ^f^	0.0209 ± 0.0018 ^a^	0.0071 ± 0.0008 ^d^	0.0006 ± 0.0002 ^c,d^	0.0847 ± 0.0041 ^b^
	**Ultrasonicated non-fermented**									
BE-ultrasound	7.8 ± 2.1 ^a^	0.8700 ± 0.0086 ^b^	0.0005 ± 0.0004 ^f^	0.0231 ± 0.0019 ^f^	0.0217± 0.0021 ^c^	0.0130 ± 0.0015 ^c^	0.0087 ± 0.0009 ^b^	0.0029 ± 0.0003 ^e^	0.0009 ± 0.0004 ^c,d^	0.0440 ± 0.0029 ^d^
Ca-ultrasound	8.3 ± 1.6 ^a^	0.7926 ± 0.0108 ^d^	0.0544 ± 0.0031 ^b^	0.0485 ± 0.0024 ^d^	0.0364 ± 0.0027 ^b^	0.0110 ± 0.0009 ^c^	0.0049 ± 0.0006 ^c^	0.0097 ± 0.0011 ^c^	0.0027 ± 0.0004 ^b^	0.0782 ± 0.0063 ^c^
Ro-ultrasound	7.8 ± 1.3 ^a^	0.8822 ± 0.0147 ^b^	0.0019 ± 0.0005 ^f^	0.0162 ± 0.0018 ^h^	0.0144 ± 0.0015 ^d^	0.0057 ± 0.0018 ^e^	0.0059 ± 0.0007 ^c^	0.0072 ± 0.0013 ^d^	0.0005 ± 0.0002 ^c^	0.0356 ± 0.0027 ^e^
	**Thermal treated and fermented with *L. uvarum*** **LUHS245 strain**									
BE-thermal-_LUHS245_	7.5 ± 0.9 ^a^	0.9442 ± 0.0132 ^a^	0.0006 ± 0.0005 ^f^	0.0120 ± 0.0015 ^i^	0.0059 ± 0.0009 ^a^	0.0057 ± 0.0011 ^e^	0.0001 ± 0.0001 ^f^	0.0006 ± 0.0004 ^f^	0.0001 ± 0.0001 ^e^	0.0166 ± 0.0017 ^g^
Ca-thermal-_LUHS245_	7.4 ± 1.1 ^a^	0.8358 ± 0.0214 ^c^	0.0060 ± 0.0007 ^e^	0.0934 ± 0.0032 ^b^	0.0042 ± 0.0006 ^a,b^	0.0090 ± 0.0010 ^d^	0.0001 ± 0.0001 ^f^	0.0166 ± 0.0014 ^a^	0.0008 ± 0.0003 ^c,d^	0.0994 ± 0.0068 ^a^
Ro-thermal-_LUHS245_	7.3 ± 1.4 ^a^	0.9385 ± 0.0219 ^a^	0.0008 ± 0.0004 ^f^	0.0278 ± 0.0019 ^g^	0.0047 ± 0.0009 ^a,b^	0.0053 ± 0.0006 ^e^	0.0001 ± 0.0001 ^f^	0.0108 ± 0.0011 ^b^	0.0004 ± 0.0002 ^e^	0.0399 ± 0.0029 ^e^
	**Ultrasonicated and fermented with *L. uvarum*** **LUHS245 strain**									
BE-ultrasound-_LUHS245_	8.9 ± 1.3 ^a^	0.8230 ± 0.0143 ^d^	0.2702 ± 0.0097 ^a^	0.1419 ± 0.0076 ^a^	0.0060 ± 0.0007 ^a^	0.0701 ± 0.0008 ^a^	0.0017 ± 0.0002 ^e^	0.0070 ± 0.0009 ^d^	0.0288 ± 0.0019 ^ª^	0.0395 ± 0.0025 ^e^
Ca-ultrasound-_LUHS245_	8.0 ± 1.2 ^a^	0.8697 ± 0.0157 ^b^	0.0013 ± 0.0005 ^f^	0.0232 ± 0.0021 ^f^	0.0217 ± 0.0019 ^c^	0.0129 ± 0.0013 ^c^	0.0086 ± 0.0014 ^b^	0.0032 ± 0.0004 ^e^	0.0007 ± 0.0004 ^c,d^	0.0446 ± 0.0032 ^d^
Ro-ultrasound-_LUHS24_	9.1 ± 0.8 ^a^	0.8408 ± 0.0139 ^c^	0.0004 ± 0.0002 ^f^	0.0228 ± 0.0023 ^f^	0.0217 ± 0.0018 ^c^	0.0227 ± 0.0019 ^b^	0.0088 ± 0.0012 ^b^	0.0033 ± 0.0005 ^e^	0.0011 ± 0.0005 ^c,d^	0.0443 ± 0.0028 ^d^

OA—overall acceptability; BE—*Boletus edulis*; Ca—*Cantharellus cibarius*; Ro—*Rozites caperata*; thermal—thermal treated by boiling for 30 min; ultrasound—ultrasonicated for 30 min; LUHS245—fermented with *L. uvarum* LUHS245 strain. Data expressed as mean values (n = 20) ± standard deviation (SD). ^a–i^ Mean values within a row with different letters are significantly different (*p* ≤ 0.05).

## Data Availability

The data are available from the corresponding author upon reasonable request.

## Supplementary Materials

The following supporting information can be downloaded at: https://www.mdpi.com/article/10.3390/foods11121800/s1, Table S1: Mushrooms volatile compound (VC) profile; Table S2: Correlations and their significance between the volatile compounds and overall acceptability of thermally treated and ultrasonicated unfermented mushrooms and between volatile compounds and the emotion ‘happy’. Table S3: Correlations and their significance between the volatile compounds and overall acceptability of thermally treated and ultrasonicated fermented mushrooms and between volatile compounds and the emotion ‘happy’.
