# Supplementary material for: Biopreservation of Wild Edible Mushrooms (Boletus edulis, Cantharellus, and Rozites caperata) with Lactic Acid Bacteria Possessing Antimicrobial Properties
Source: Foods. 2022 Jun 18;11(12):1800. doi: 10.3390/foods11121800 (PMC9223197; doi:10.3390/foods11121800)
Supplement: Supplementary file 1 [file foods-11-01800-s001.zip › foods-1755535-supplementary.pdf]

Supplementary materials include the following:

Total pages: 10; total tables: 3;

Table S1 Mushrooms volatile compound (VC) profile.

Table S2 Correlations and their significance between the volatile compounds and overall acceptability of thermally treated and ultrasonicated unfermented mushrooms and between volatile compounds and the emotion ‘happy’.

Table S3 Correlations and their significance between the volatile compounds and overall acceptability of thermally treated and ultrasonicated fermented mushrooms and between volatile compounds and the emotion ‘happy’.

Table S1. Volatile compound (VC) profile (RT – retention time; BE - *Boletus edulis*; Ca – *Cantharellus*; Ro - *Rozites caperata;* VC – volatile compound; LUHS245 – fermented with *L. uvarum* LUHS245 strain; thermal – thermal treated by boiling 30 min; ultrasound – ultrasonicated for 30 min. Data are represented as means (n = 3, replicates of analysis).

RT, min	VC	BE - nontreated	Ca - nontreated	Ro - nontreated	BE-thermal	Ca-thermal	Ro-thermal	BE-ultrasound	Ca-ultrasound	Ro-ultrasound	BE-thermal-LUHS245	Ca-thermal-LUHS245	Ro-thermal-LUHS245	BE-ultrasound-LUHS245	Ca-ultrasound-LUHS245	Ro-ultrasound-LUHS245
2,375	Acetic acid	0	0	0	0	0	0	0	0	0	0	0	2,322	0	0	0
3,024	3-methyl-butanal	0,572	0	0,487	0	0	0	0	0	0	0	0	0	0	0	0
3,779	Acetoin	0	0	0	0	0	0	0	0	0	0	14,84	2,684	2,763	0	0
4,355	3-methyl-1-butanol	0,567	0	0	0	0	0	0	0	0	0	12,98	0	18,12	1,856	6,981
5,277	2,3-Butanediol	0	0	0	0	0	0	0	0	0	0	0	0	3,727	0	0
5,763	Hexanal	0	1,886	0,445	0,922	5,323	0,986	0	3,461	0,618	0	0	16,84	0	3,164	1,689
6,401	1,3-Octadiene	0	0,299	0	0	0,91	0	0	0,463	0	0	0	0	0	0	0
6,915	2-Butenoic acid ethyl ester	0	0	0	0	0	0	0	0	0	0	0	0	0	0	0,241
7,164	3-methyl-butanoic acid ethyl ester	0	0	0	0	0	0	0	0	0	0	0	0	0	0	2,123
7,567	1-Hexanol	0	0	0	0	0	0,257	0	0	0	0	0	1,094	0	2,715	5,078
8,11	2-Heptanone	0	0	0	1,047	0	0	0	0	0	1,833	0	0	0	0	0,312
8,424	Heptanal	0	0	0	2,061	0,357	0	1,895	0,323	0	0	0	0	0	0	0
9,451	Ethyl tiglate	0	0	0	0	0	0	0	0	0	0	3,308	0	2,899	0	14,1
9,631	2,7-dimethyl-4,5-octanediol	0	0	0	0	0	0	0	0	0	0	1,457	0	0	0	0
9,862	1-methyl-2-(3-methylpentyl)-cyclopropane	0	0	0	0	0	0	0	0	0	0	0	0	0	0	0,034
9,865	2-Heptenal	0	0,209	0	0	0,554	0	0	0,394	0	0	0,545	9,017	0	0,456	0
9,949	Benzaldehyde	0,538	1,267	35,6	4,527	1,965	44,45	1,528	2,224	51,6	4,668	1,646	0	0	1,746	6,316
10,244	Heptyl formate	0	0	0	0	0	0	0	0	0	0	0	0	0	1,113	0
10,368	Isopropyl tiglate	0	0	0	0	0	0	0	0	0	0	0	0	0	0	0,746
10,493	1-Octen-3-ol	61,86	55,22	50,4	57,87	51,13	35,87	63,63	50,92	31,74	52,72	22,21	7,304	26,76	36,47	11,1
10,584	2,5-Octanedione	0	0	0	0	0	0	0	0	0	0	0	3,394	0	0	0
10,669	3-Octanone	5,621	0	1,814	0	0	4,568	0	0	4,351	0	7,32	0	26,4	12,79	12,5
10,812	2-pentylfuran	0	0	0	0	0,12	0	0	0,065	0	0	7,093	3,528	0	0	0
10,885	3-Octanol	8,814	0,525	3,713	7,003	1,287	6,082	0	0,602	4,201	0	0	3,258	9,115	7,463	5,46

10,988	Hexanoic acid ethyl ester	0	0	0	0	0	0	0	0	0	0	0	0	0	0	5,235
11,091	Octanal	5,474	0,367	1,34	1,267	0,651	1,513	1,35	0,436	1,49	1,501	0	2,819	0	1,716	0
11,145	α-Phellandrene	0	0	0	0,626	0	0	0	0	0	0	0	0	0	0	0
11,344	Acetic acid hexyl ester	0	0	0	0	0	0	0	0	0	0	0	0	0	0	0,071
11,673	p-Cymene	0	0	0	0,955	0	0	1,482	0	0	0,961	0,105	0	0	0,079	0,031
11,788	Limonene	0,052	0	0,065	3,55	0	0	4,595	0	0,409	3,642	0,587	0	0,151	0	0,188
11,844	3-ethyl-2-methyl-1,3-hexadiene	0	1,816	0	0	2,795	0	0	3,1	0	0	0	2,034	0	3,316	0
11,883	Benzyl alcohol	0	0	0,071	0	0	0	0	0	0	0	0	0	0	0	3,108
11,995	3-Octen-2-one	0	0	0	0	0,577	0,054	0	0	0,058	0	0	0,757	0	0	0
12,04	2-(1-pentenyl)furan	0	0	0	0	0	0	0	0	0	0	0,724	0	0	0	0
12,133	Benzeneacetaldehyde	1,754	0	0	0	0	0	0,536	0	0	0	0	0	0	0	0
12,172	1-ethyl-1-methyl-cyclopentane	0	0,716	0	0	0	0	0	0	0	0	0	0	0	0	0
12,195	Sulfurous acid dicyclohexyl ester	0	0	0,133	0	0	0	0	0	0	0	0	0	0	0	0
12,227	4,6-dimethyl-2-heptanone	0	0	0	0	0	0	0	0	0	0	0	0	0	0	0,673
12,476	Oct-(2E)-enal	4,551	16,34	2,247	3,157	22,2	2,207	4,445	20,72	2,504	4,32	1,518	9,941	0,32	7,662	2,056
12,728	(E)-2-octen-1-ol	0	16,91	1,129	9,847	4,538	0,519	11,99	11,28	0,213	9,622	3,584	4,228	0	1,984	2,996
12,788	1-Octanol	8,01	0	0	0	0	0	0	0	0	0	0	0	2,526	11,55	0
12,953	(5-Ethylcyclopent-1-enyl)methanol	0	0	0	0	0	0	0	0	0	0	0	0	0	0,155	0
13,01	3-ethyl-2,5-dimethylpyrazine	0	0	0	0	0	0	0	0	0	0	0	0	0,296	0	0
13,011	1-Nonen-3-ol	0	0,011	0	0	0	0	0	0	0	0	0	0	0	0	0
13,089	5-Nonen-2-one	0	0	0	0	0	0	0	0	0	0	0,444	0	0	0	0
13,119	1-Adamantanol	0	0	0	0,314	0	0	0,43	0	0	0,596	0	0	0	0	0
13,267	2-iodo-3-methyl-butane	0	0	0	0	0	0	0	0	0	0	0	0	1,071	0	0
13,314	2-Nonanone	0	0	0	0	0	0	0	0	0	4,138	4,969	4,162	0	0	4,983
13,341	6-Methyl-hept-2-en-4-ol	0	0,543	0	0	1,399	0	0	0,877	0	0	0	0	0	0	0
13,518	2-Nonanol	0	0	0	0	0	0	0	0	0	0	0	0,689	0,393	0	0
13,612	Nonanal	0,286	0,453	0,3	1,157	0,907	0,962	1,476	0,641	0,718	1,438	1,097	4,389	0	0,642	0,918

13,716	(E,E)-2,4-octadienal	0	0,115	0	0	0,225	0	0	0	0	0	0,758	0	0,073	0	0
13,865	Phenylethyl Alcohol	0,237	0	0	0	0	0	0	0	0	0	1,439	0,593	1,799	0,803	2,539
13,945	N-hexyl-1-hexanamine	0	0	0	0	0	0	0	0	0	0	0	0	1,64	0	0
14,083	Methyl octanoate	0	0	0	0	0	0	0	0	0	0	0,183	0	0	0	0
14,311	N-(3-Methylbutyl)acetamide	0	0	0	0	0	0	0	0	0	0	0	0	0,098	0	0
14,317	Pentyl propyl ketone	0	0,041	0	0	0,046	0	0	0,042	0	0	0,348	0	0	0	0
14,435	trans-3-Nonen-2-one	0	0	0,013	0	0	0	0	0	0,027	0	0	0,162	0	0	0,372
14,613	7-methyl-4-octanol	0	0,541	0	0	0,333	0	0	0,395	0	0	0	0	0	0	0
14,922	(E)-non-2-enal	0	0,638	0,047	0,181	0,941	0,134	0,238	0,804	0,12	0,327	0,571	1,962	0	0,844	0,252
14,96	2,3,5-Trimethyl-6-ethylpyrazine	0	0	0	0	0	0	0	0	0	0	0	0	0,127	0	0
15,042	Acetic acid phenylmethyl ester	0	0	0	0	0	0	0	0	0	0	0	0	0	0	0,091
15,074	4-ethylphenol	0	0	0	0	0	0	0	0	0	0	0	0	0,373	0	0
15,112	Octanoic acid	0	0,115	0,06	0,19	0,102	0,08	0,315	0,075	0,046	0,608	1,488	0	0	0	0
15,182	1-Nonanol	0,243	0	0	0	0	0,064	0	0	0	0	0	0	0,11	0,307	0,86
15,227	2-Piperidinone	0	0	0,025	0	0	0	0	0	0	0	0	0	0	0	0
15,274	1-Nonen-4-ol	0	0,041	0	0	0	0	0	0,071	0	0	0	0	0	0	0,527
15,577	Naphthalene	0	0,045	0,028	0	0	0,015	0	0,064	0,031	0	0	0	0	0	0
15,611	3,6-Dimethyl-2,3,3a,4,5,7a-hexahydrobenzofuran	0,088	0	0	1,408	0	0	1,526	0	0	1,04	0	0	0	0	0
15,675	2-Decanone	0	0	0	0	0	0	0	0	0	0	0,117	0	0,058	0	0
15,716	3-butylcyclohexene	0	0	0,029	0	0,112	0,046	0	0,107	0,066	0	0	0,628	0	0	0
15,753	Octanoic acid ethyl ester	0	0	0	0	0	0	0	0	0	0	0,56	0	0,154	0	1,343
15,84	Dodecane	0,014	0	0,021	0,149	0,065	0,032	0,118	0	0,043	0,188	0	0	0	0	0
15,975	Decanal	0,158	0,106	0,026	0	0,106	0,085	0,171	0,115	0,09	0,248	0	0,263	0	0,126	0,105
16,171	(E,E)-2,4-nonadienal	0,02	0,192	0,101	0	0,38	0	0	0,272	0,205	0,129	0	2,265	0,029	0,179	0,248
16,253	2-methyl-5-(1-methylethenyl)cyclohexanol	0	0	0	0	0	0,017	0	0	0,028	0	0	0	0	0	0
16,305	Phenoxyethanol	0	0,01	0	0	0	0	0	0	0	0	0	0	0	0	0

16,416	2,6,6-trimethyl-1-cyclohexene-1-carboxaldehyde	0	0	0	0	0,382	0	0	0,414	0	0	1,257	0	0	0,37	0
16,49	Benzothiazole	0	0,029	0	0	0	0,016	0	0,022	0,034	0	0	0	0	0,051	0
16,655	2-methylbutanoic acid hexyl ester	0	0	0	0	0	0	0	0	0	0	0	0	0	0	0,109
16,731	Heptylidene acetone	0	0	0	0,663	0	0	1,067	0	0	0,576	0	0	0,062	0	0
16,991	Isopentyl hexanoate	0	0	0	0	0	0	0	0	0	0	0	0	0	0	0,095
17,066	β-Ethylphenethyl alcohol	0,04	0	0	0	0	0	0	0	0	0	0	0	0,093	0	0
17,099	1,3-bis(1,1-dimethylethyl)benzene	0	0	0,039	0,258	0,095	0,071	0,165	0,05	0,071	0,472	0,218	0,14	0	0,085	0
17,206	Dec-(2E)-enal	0,027	0,06	0,035	0	0,087	0,125	0	0	0,096	0	0	1,661	0	0	0,276
17,255	2,6,6-trimethyl-1-cyclohexene-1-acetaldehyde	0	0	0	0	0	0	0	0,127	0	0	0	0	0	0	0
17,275	Vinyl caprylate	0	0	0	0	0	0	0	0	0	0	0	0	0	0	0,238
17,293	Nonanoic acid	0,315	0,223	0,277	0,828	0,477	0,195	0,804	0,135	0,128	2,373	3,409	1,96	0,17	0,3	0
17,415	Dipentyl ketone	0	0	0	0	0	0	0	0	0	0	0	0	0	0	0,292
17,432	1-Decanol	0	0	0	0	0	0	0	0	0	0	0	0	0,083	0	0
17,502	2-phenyl-crotonaldehyde	0,382	0	0,022	0,188	0	0,027	0,233	0	0,035	0,206	0	0	0	0	0
17,545	(Z)-3-Octen-1-ol acetate	0	0	0	0	0	0	0	0	0	0	0,18	0	0	0	0
17,585	2,2,4,15,17,17-hexamethyl-7,12-bis(3,5,5-trimethylhexyl)octadecane	0	0,119	0	0	0	0	0	0	0	0	0	0	0	0	0
17,605	2,2,4,10,12,12-hexamethyl-7-(3,5,5-trimethylhexyl)-6-Tridecene	0	0	0	0	0,08	0	0	0,095	0	0	0	0	0	0	0
17,614	4,6-dimethyldodecane	0,021	0	0	0,101	0	0,038	0,121	0	0,017	0,129	0	0	0	0	0
17,617	5-methyl-2-(1-methylethyl)-1-hexanol	0,023	0	0	0	0	0	0	0	0	0	0	0	0	0	0,108
17,619	2,6,10-trimethyldodecane	0	0	0	0	0	0	0	0	0	0	0,328	0	0	0	0
17,888	2-Undecanone	0,054	0	0,26	0	0,297	0,511	0,67	0,264	0,476	5,09	1,14	4,621	0,25	0,574	3,438
17,96	Indole	0	0	0,016	0	0	0,017	0	0	0,024	0	0	0	0	0	0
18,017	Methyl nonyl carbinol	0	0	0,049	0	0	0,038	0	0	0,041	0	0	0,356	0,063	0	0,495
18,18	Undecanal	0,016	0,014	0	0	0	0,028	0	0,013	0,027	0	0	0,06	0	0	0

18,376	Deca-(2E,4E)-dienal	0,032	0,215	0,074	0,116	0,211	0,289	0	0,215	0,179	0	0	1,126	0,02	0,215	0,287
18,663	Methyl 8-oxooctanoate	0	0,019	0	0	0	0	0	0,023	0	0	0	0	0	0	0,148
18,706	3-[(1Z)-1,3-Butadienyl]-4-vinylcyclopentene	0	0	0	0	0	0	0	0	0	0	0,156	0	0	0	0
19,203	Propanoic acid, 2-methyl-, 2,2-dimethyl-1-(2-hydroxy-1-methylethyl)propyl ester	0	0,009	0,032	0,331	0	0	0,278	0	0	0,189	0	0	0,038	0	0
19,288	n-Decanoic acid	0,032	0	0,03	0	0	0	0	0	0	0,22	0	0,299	0	0	0
19,334	2-Undecenal	0	0,092	0	0	0,115	0,105	0	0,097	0,092	0	0	0,992	0	0,21	0
19,359	5-heptyldihydro-2(3H)-furanone	0,056	0	0	0	0	0	0	0	0	0	0	0	0	0	0,412
19,493	6-Dodecanone	0	0	0	0	0	0	0	0	0	0	0	0	0	0	0,249
19,644	Propanoic acid, 2-methyl-, 3-hydroxy-2,4,4-trimethylpentyl ester	0	0	0	0,385	0	0	0	0	0	0,617	0	0	0	0	0
19,656	trans-4,5-Epoxy-(E)-2-decenal	0	0,041	0	0	0,134	0	0	0,05	0	0	0	1,332	0	0	0
19,811	3-methyl-N-(2-phenylethylidene)-1-butanamine	0,018	0	0,976	0,179	0	0,343	0	0	0,074	0	0	0	0	0,078	0,333
20,044	Tetradecane	0,022	0,019	0,011	0,117	0	0,031	0,135	0	0,032	0,202	0,1	0,088	0,028	0,04	0
20,166	2-butyl-1-octanol	0	0,007	0	0	0	0	0	0	0	0	0	0	0	0	0
20,222	Isophytol	0	0	0,021	0	0	0,055	0	0	0	0	0	0	0	0	0,129
20,363	2,4,7,9-Tetramethyl-5-decyn-4,7-diol	0	0	0	0	0	0,043	0,14	0	0,023	0	0,084	0,131	0	0	0
20,704	9-oxo-nonanoic acid methyl ester	0	0,051	0	0	0	0,018	0	0,05	0,013	0	0	0	0	0	0
20,813	Trans-Tetradec-2-enal	0	0	0	0	0	0,032	0	0	0	0	0	0	0	0	0
20,834	(Z)-octadec-9-enal	0	0	0	0	0	0	0	0	0	0	0	0,316	0	0	0
20,932	4-(2,6,6-trimethyl-1-cyclohexen-1-yl)-2-butanone,	0	0,039	0	0	0	0	0	0	0	0	0	0	0	0	0
21,125	6,10-dimethyl-5,9-undecadien-2-one	0,017	0,061	0,017	0,055	0,09	0,022	0,077	0,073	0,019	0,078	0,269	0,065	0,031	0,173	0,035
21,197	2-methylpentanoic acid anhydride	0	0	0	0	0	0	0	0,01	0	0	0	0	0	0	0

21,238	β-Barbatene	0	0	0,027	0	0	0,034	0	0	0,054	0	0	0,193	0	0	0
21,466	Dodecanol	0	0,035	0	0	0	0	0	0	0	0	0	0	0	0	0
21,48	9-Decen-1-yl acetate	0	0	0	0	0	0	0	0	0	1,275	0,209	0,596	0	0	0
21,487	n-Tridecan-1-ol	0	0	0	0	0	0	0,06	0	0	0	0	0	0	0	0
21,648	(1-methyl-3-butenyl)benzene	0	0	0	0	0	0	0	0	0	0	0	0	0,092	0	0
21,88	4-(2,6,6-trimethyl-1-cyclohexen-1-yl)-3-buten-2-one	0	0,542	0	0	0,742	0	0	0,821	0	0	2,291	0	0	0,725	0
22,052	Benzyl tiglate	0	0	0	0	0	0	0	0	0	0	0	0	0	0	0,077
22,258	2,4-bis(1,1-dimethylethyl)phenol	0,036	0,018	0	0,471	0,03	0,022	0,418	0,021	0,01	0,485	0,129	0	0	0	0
22,351	Butylated Hydroxytoluene	0	0	0	0	0	0	0	0	0	0,112	0	0	0	0	0
22,623	4-(1-piperidinyl)-2-butanone	0	0	0	0	0,147	0	0	0	0	0	0	0	0	0	0
22,751	Hentriacontane	0,015	0	0	0	0,022	0	0,106	0	0	0	0,128	0	0	0	0
22,819	5,6,7,7a-tetrahydro-4,4,7a-trimethyl-2(4H)-Benzofuranone	0	0	0	0	0,028	0	0	0,061	0	0	0,213	0	0	0,038	0
23,051	Dodecanoic acid	0,032	0	0,02	0,075	0,046	0	0	0	0	0	0	1,648	0,036	0	0
23,676	2-Pentylcyclopentanone	0	0	0	0	0	0	0	0	0	0	0	0,083	0	0	0
23,685	Ethyl dodecanoate	0	0	0	0	0	0	0	0	0	0	0	0	0,03	0	0

RT – retention time; BE - *Boletus edulis*; Ca – *Cantharellus*; Ro - *Rozites caperata;* VC – volatile compound; LUHS245 – fermented with *L. uvarum* LUHS245 strain; thermal – thermal treated by boiling 30 min; ultrasound – ultrasonicated for 30 min. Data are represented as means (n = 3, replicates of analysis).

Table S2. Correlations and their significance between thermal treated and ultrasonicated nonfermented mushrooms volatile compound and overall acceptability and between volatile compound and emotion ‘happy’.

VC	OA		Happy	
	Pearson Correlation	Sig. (2-tailed)	Pearson Correlation	Sig. (2-tailed)
Hexanal	0.385	0.114	0.619^{**}	0.006
2-Heptanone	-0.101	0.690	-0.088	0.728
Heptanal	-0.041	0.873	-0.202	0.422
Benzaldehyde	-0.103	0.685	-0.472^{*}	0.048
1-Octen-3-ol	0.244	0.329	0.190	0.449
3-Octanone	-0.103	0.683	-0.472^{*}	0.048
3-Octanol	-0.134	0.596	-0.416	0.086
Octanal	-0.086	0.735	-0.862^{**}	0.0001
p-Cymene	-0.088	0.728	-0.358	0.145
Limonene	-0.099	0.695	-0.380	0.120
3-ethyl-2-methyl-1,3-hexadiene	0.317	0.199	0.841^{**}	0.0001
Oct-(2E)-enal	0.367	0.134	0.778^{**}	0.0001
(E)-2-octen-1-ol	0.082	0.747	0.457	0.057
6-Methyl-hept-2-en-4-ol	0.370	0.130	0.628^{**}	0.005
Nonanal	0.121	0.632	-0.530^{*}	0.024
3,6-Dimethyl-2,3,3a,4,5,7a-hexahydrobenzofuran	-0.088	0.727	-0.335	0.174
Heptylidene acetone	-0.082	0.747	-0.358	0.144

VC – volatile compound; OA – overall acceptability; * - Correlation is significant at the 0.05 level (2-tailed); * - Correlation is significant at the 0.01 level (2-tailed).

Table S3. Correlations and their significance between thermal treated and ultrasonicated fermented mushrooms volatile compound and overall acceptability and between volatile compound and emotion 'happy'.

VC	OA		Happy	
	Pearson Correlation	Sig. (2-tailed)	Pearson Correlation	Sig. (2-tailed)
Acetic acid	-0.245	0.328	-0.204	0.418
Acetoin	-0.227	0.365	-0.033	0.897
3-methyl-1-butanol	0.335	0.175	0.755**	0.0001
2,3-Butanediol	0.385	0.141	0.999**	0.0001
Hexanal	-0.239	0.341	-0.274	0.271
3-methyl-butanoic acid ethyl ester	0.482	0.081	-0.279	0.334
1-Hexanol	0.379	0.121	-0.359	0.143
2-Heptanone	-0.088	0.728	-0.243	0.332
Ethyl tiglate	0.464	0.053	-0.045	0.860
2,7-dimethyl-4,5-octanediol	-0.218	0.386	-0.181	0.473
2-Heptenal	-0.268	0.282	-0.229	0.360
Benzaldehyde	0.189	0.483	-0.563*	0.023
Heptyl formate	0.020	0.937	-0.201	0.424
1-Octen-3-ol	-0.078	0.758	0.020	0.937
2,5-Octanedione	-0.245	0.328	-0.204	0.418
3-Octanone	0.543*	0.020	0.819**	0.0001
2-pentylfuran	-0.336	0.173	-0.275	0.269
3-Octanol	0.519*	0.027	0.622**	0.006
Hexanoic acid ethyl ester	0.423	0.080	-0.205	0.413
Octanal	-0.322	0.192	-0.420	0.082
Limonene	-0.179	0.477	-0.210	0.402
3-ethyl-2-methyl-1,3-hexadiene	-0.129	0.610	-0.307	0.216
Benzyl alcohol	0.425	0.079	-0.205	0.414
Oct-(2E)-enal	-0.260	0.297	-0.518*	0.028
(E)-2-octen-1-ol	-0.286	0.250	-0.564*	0.015
1-Octanol	0.076	0.766	0.017	0.946
2-iodo-3-methyl-butane	0.360	0.143	0.999**	0.0001
2-Nonanone	-0.135	0.594	-0.617**	0.006
Nonanal	-0.314	0.205	-0.452	0.060
Phenylethyl Alcohol	0.567*	0.014	0.326	0.187
N-hexyl-1-hexanamine	0.359	0.144	0.999**	0.0001
(E)-non-2-enal	-0.319	0.197	-0.461	0.054
3,6-Dimethyl-2,3,3a,4,5,7a-hexahydrobenzofuran	-0.183	0.468	-0.205	0.415
Octanoic acid ethyl ester	0.381	0.118	-0.170	0.500
(E,E)-2,4-nonadienal	-0.203	0.419	-0.252	0.313
2,6,6-trimethyl-1-cyclohexene-1-carboxaldehyde	-0.211	0.400	-0.243	0.331
Dec-(2E)-enal	-0.179	0.477	-0.243	0.332
Nonanoic acid	-0.459	0.055	-0.401	0.099
Deca-(2E,4E)-dienal	-0.141	0.576	-0.295	0.235
trans-4,5-Epoxy-(E)-2-decenal	-0.246	0.324	-0.204	0.418
9-Decen-1-yl acetate	-0.337	0.171	-0.335	0.175
4-(2,6,6-trimethyl-1-cyclohexen-1-yl)-3-buten-2-one	-0.213	0.397	-0.247	0.323
Dodecanoic acid	-0.241	0.335	-0.183	0.468

VC – volatile compound; OA – overall acceptability; * - Correlation is significant at the 0.05 level (2-tailed);
* - Correlation is significant at the 0.01 level (2-tailed).
